# Diathesis stress or differential susceptibility? testing the relationship between stressful life events, neuroticism, and internet gaming disorder among Chinese adolescents

**DOI:** 10.1371/journal.pone.0263079

**Published:** 2022-01-27

**Authors:** Hao Li, Xiong Gan, Xin Li, Ting Zhou, Xin Jin, Congshu Zhu

**Affiliations:** 1 Department of Psychology, College of Education and Sports Sciences, Yangtze University, Jingzhou, China; 2 Xian Tao No.1 Middle School, XianTao, China; 3 Yangtze University College of Technology and Engineering, Jingzhou, China; The University of Melbourne, AUSTRALIA

## Abstract

A previous study has documented that stressful life events is positively related to Internet gaming disorder (IGD) among adolescents. However, the mechanism underlying this relationship remains unclear. The current study examined whether the link between stressful life events and adolescent IGD was moderated by neuroticism and whether the interaction of stressful life events and neuroticism supported the diathesis stress model or differential susceptibility model. To this end, self-report questionnaires were distributed. Participants were 927 Chinese adolescents (mean_age_ = 14.53 years, 51.02% male). After controlling for adolescent gender, age, family economic situation, and family socioeconomic status, the results revealed that stressful life events could be positively associated with adolescent IGD and that this link is moderated by neuroticism. Moreover, the results of interaction effects supported the "diathesis stress" model. The above findings contributed to a better understanding of how and when stressful life events increase the risk of IGD and provided new evidence for the diathesis stress hypothesis.

## 1. Introduction

As of June 2021, the number of Internet users in China has reached 1 billion, while the number of online game users has reached 510 million, including more than 100 million young users [1]. Internet gaming disorder (IGD) is defined as a persistent and recurrent engagement in Internet gaming which results in distress [2]. As IGD has become a global public health issue, it has been included in the updated version of the Diagnostic and Statistical Manual Disorders (DSM-5) and the International Classification of Diseases (ICD-11) and is thus attracting enormous attention from researchers [3, 4]. Due to the differences in measurement tools, the rates of possible IGD among adolescents in China ranged between 2% and 17% [5, 6]. IGD has serious adverse effects on youth and adolescents because it not only leads to a variety of externalizing problems (e.g., academic dysfunction, poor sleep quality, substance use) but also causes internalizing problems (e.g., anxiety, depression) among adolescents [7, 8]. Therefore, it is urgent to examine risk factors and mechanisms that contribute to the development of IGD to provide a scientific basis for its intervention practice.

Among the many factors associated with IGD, stressful life events have attracted much attention of researchers [9, 10]. Based on the general strain theory, a variety of strain or stress experienced by adolescents might cause negative emotions, which subsequently cause problem behaviors such as Internet addiction [11]. Theorists have also proposed that IGD is a behavioral response to pre-existing stressful life events such as family conflict, academic underachievement, and peer rejection [11, 12]. Specifically, stressful life events can cause a variety of psychological strains and negative emotions in an individual, and Internet gaming can become a psychological escape that distracts a user from a real-life problem or allows them to vent negative emotions [11]. Previous studies have demonstrated that excessive online gaming may be a coping strategy for life problems [13]. And another study also discovered a positive relationship between stressful life events and problematic Internet use [14]. Consequently, the present study investigates the relationship between stressful life events and adolescent IGD and puts forward the hypothesis that stressful life events can positively correlate with adolescent IGD.

### 1.1. The moderation effect of neuroticism: "Diathesis stress model" or "differential susceptibility"

Neuroticism is an emotion-related personality trait that reflects differences in individual emotional stability [15]. The results of several studies indicate that the response of the low neurotic population to stress is mild and slow, while the high neurotic population has the characteristics of being emotional, impulsive, dependent, and poor self-feeling [16, 17]. The theory of addictions highlights personality vulnerabilities that interact with stress in the development of addiction [18], and neuroticism can moderate perception of stress and its consequences [19]. Previous studies also investigated that high neuroticism was positively associated with high adolescent problematic gaming behavior [20] and neuroticism is a potential predictor of Internet gaming disorder [21, 22]. Thus, the interaction between neuroticism and stressful life events could be crucial in the development of IGD among adolescents.

**Fig 1:**
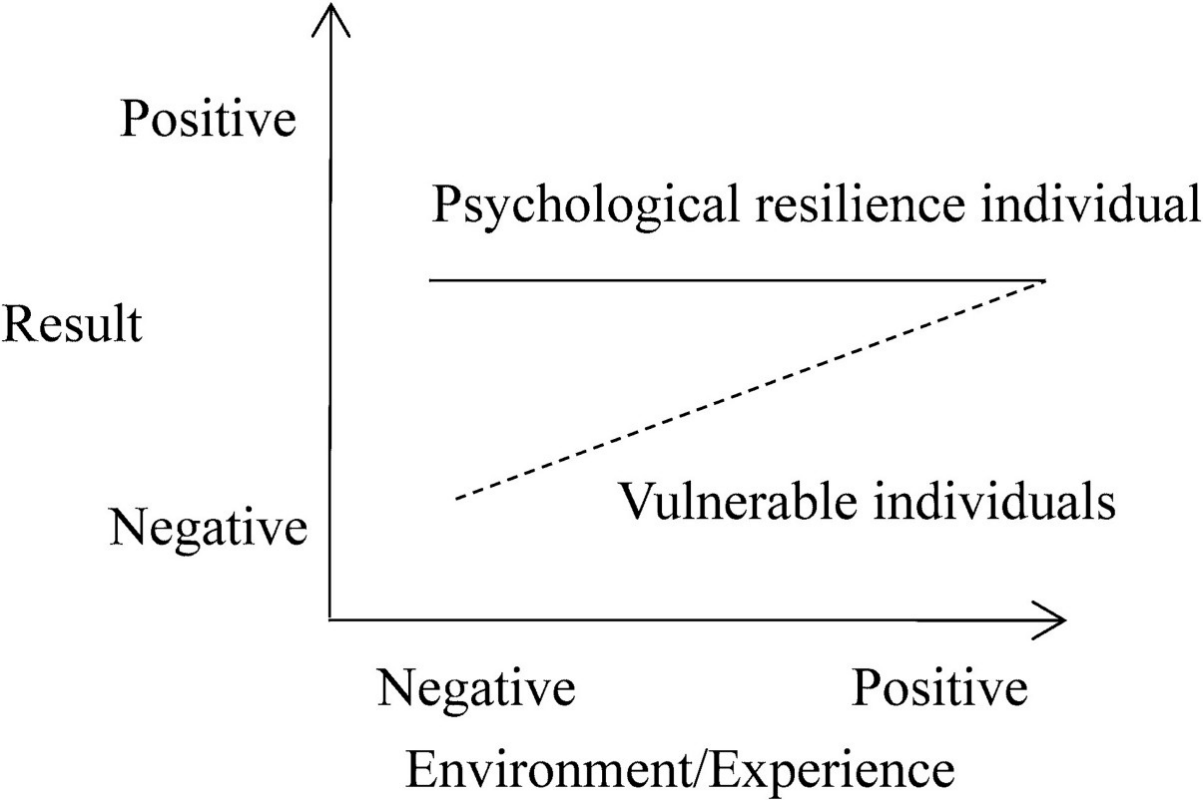
Diathesis-stress model.

**Fig 2:**
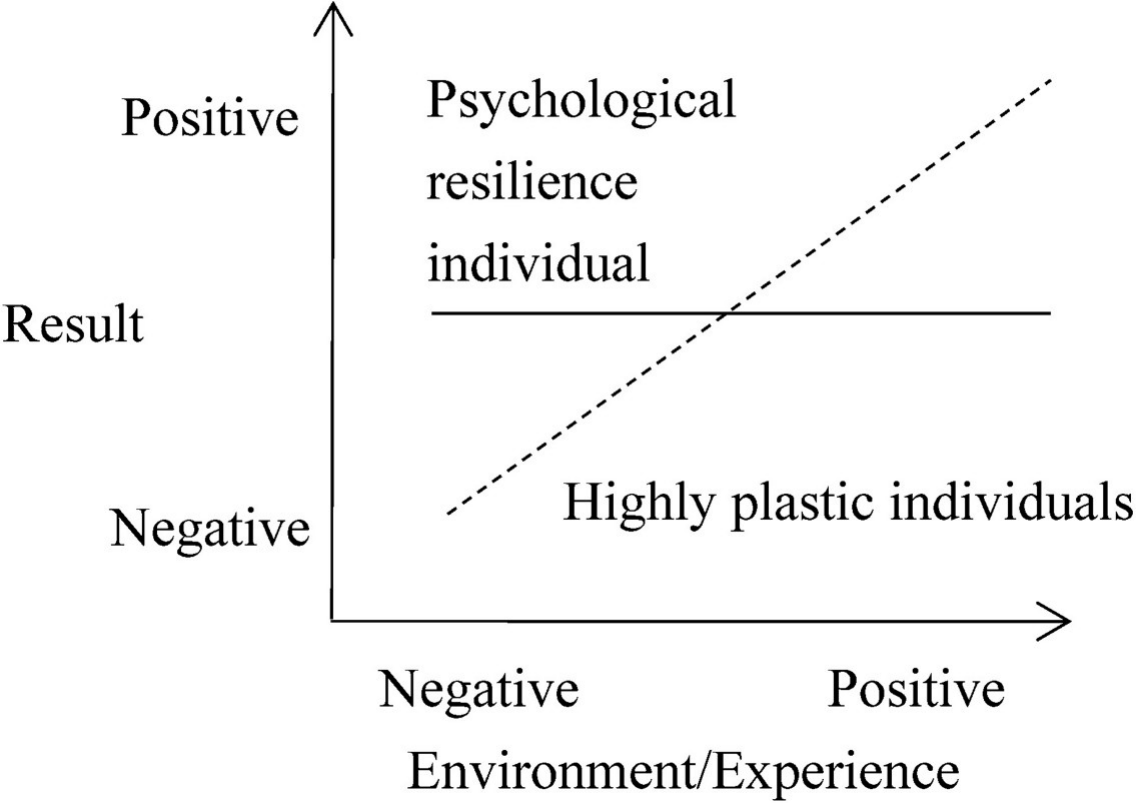
Differential susceptibility model.

Two theories that have attracted much attention can be used to explain the interaction between personality (neuroticism) and the environment (stressful life events). The Diathesis-Stress Model assumes that there is an interaction between stressors and personality traits, such that personality vulnerability increases the likelihood of psychopathology only when combined with the experience of significant stressors [23]. In other words, due to their own "vulnerability" factors (such as temperament, physiology, or genes), certain individuals are more vulnerable to the adverse impact of stressful life events and show negative development results (e.g., Internet gaming disorder) (see Fig 1). But on the other hand, the diathesis-stress framework implies that there is no difference in the performance of different individuals who are vulnerable or resilient in a positive environment [24]. The Differential Susceptibility Model believes that personal development is plastic rather than fragile. An individual’s "vulnerability factor" should be regarded as a "plasticity factor" of development, which makes some individuals more malleable or susceptible than others to both negative and positive environmental influences [25]. That is to say, the environment affects the development of highly plastic individuals in a better or worse way (see Fig 2).

Which model does the interaction between neuroticism and stressful life events most consistently match with? The results of previous studies are not consistent. Wang and his colleagues found that in a negative environment, individuals with high neuroticism are more likely to show anxiety symptoms when they encounter stressful life events [17]. The results support the diathesis-stress model. However, in subsequent research, Rioux revealed that individuals with certain genetic traits are not only susceptible to positive situations and can adapt well to the environment, but also more vulnerable to negative situations and suffer from maladaptive behaviors. The results better support the differential susceptibility model [26]. It is still debatable whether adolescent neuroticism should be regarded as “vulnerability” or “plasticity”.

### 1.2. The present study

In summary, the current study aimed to examine how stressful life events was associated with adolescent IGD, tested the moderator role of neuroticism, and provided valuable information for early prevention and intervention of adolescent IGD, all within the framework of general strain theory and addiction theory [11, 18]. Then, we investigated whether the interaction of stressful life events and neuroticism supported the diathesis-stress model or the differential susceptibility model, based on the discrepancy of prior study results [17, 26]. Different moderation models have different practical implications, so it is very critical to verify which moderation model the above relationship belongs to.

## 2. Methods

### 2.1. Participants and procedures

The present study was approved by the Institutional Review Board of Yangtze University. The data was collected from three public middle schools in Jingzhou (a city in central China) by the convenient sampling method. After obtaining informed consent from the participants and their parents or legal guardians involved, the research was conducted in participants’ classrooms by trained senior students majoring in psychology during class time. All participants were told that their answers would be protected and that they were free to withdraw from the study at any time. In this study, a total of 1400 questionnaires were distributed, and 1249 were recovered, of which 927 were valid, and the effective recovery rate was 66.21%. There were 473 boys and 454 girls, with an average age of 14.53 ± 1.86 years (range = 11–20).

### 2.2. Measures

#### 2.2.1. Internet gaming disorder

IGD was assessed by the Chinese version [27] of Pathological Video-Game Use Questionnaire [28]. Participants answered 11 items on a 3-point scale (0 = never, 1 = sometimes, 2 = yes). Example items included ‘‘Have you tried to play online games less often or for shorter periods, but are unsuccessful?” Then the answers were recoded (0 = never, 0.5 = sometimes, 1 = frequently), and the total score was calculated for each participant. A higher mean score indicated a stronger tendency of IGD, and the cut-off score for identifying adolescents with IGD was 5 or more [29]. This measure had demonstrated good reliability and validity in Chinese adolescents [30, 31]. Cronbach’s alpha for this scale in the present study was 0.75.

#### 2.2.2. Neuroticism

The study selected the Chinese version of the Big Five personality revised by Costa and McCrae [32]. A sample item was“Sometimes I feel angry and resentful.” Adolescents were asked to answer 12 items on a 5-point scale ranging from 1 (strongly disagree) to 5 (strongly agree). Mean scores for each dimension were calculated, with higher scores indicating higher levels of neuroticism. This measure has demonstrated good reliability and validity among Chinese adolescents [33]. Cronbach’s alpha for this scale in the present study was 0.83.

#### 2.2.3. Stressful life events

Stressful life events were measured using the Adolescent Self-Rating Life Events Check List (ASLEC) by Liu et al. [34]. The questionnaire consisted of 27 items and measured life stress from four sources, including physical disease (e.g., personal serious illness or injury), family (e.g., death of relatives), school (e.g., examination pressure), and interpersonal relations (e.g., break-up with close friends). Respondents were asked to rate whether each event had occurred in the past half year on a scale of 0 to 5 (0 = did not occur; 5 = occurred and was extremely stressful). The total score was calculated for each participant, with higher scores indicating more life events the subject experienced. This measure has demonstrated good reliability and validity among Chinese adolescents [35, 36]. Cronbach’s alpha for this scale in the present study was 0.93.

### 2.3. Statistical analysis

All the statistical analyses were conducted with Mplus. First, hierarchical regression is used to test the interaction between stressful life events and neuroticism on the IGD for adolescents, and then to further probe the interactions to determine whether they are more consistent with diathesis-stress or differential susceptibility models. Traditionally, researchers have detected the influence of independent variables on dependent variables at different levels of moderator variables by conducting a simple slopes analysis. But it only focuses on teenagers with plus or minus one standard deviation and estimates the differences in behavior or psychological development between a high neurotic group (M+1SD) and a low neurotic group (M-1SD) of adolescents in different environments. The present study examines specific models of significant interactions using the Analysis of Region of Significance [37], which examines all the values of moderator variables when independent variables are significantly associated with dependent variables.

When using the RoS method to test the diathesis-stress model and differential susceptibility model, the following 4 conditions should be met [37]: (1) Determine regions of significance on the independent (X) variable (RoS on X). Specifically, to identify the value range of X for which adolescents with different levels of neuroticism (Z) and outcome variables (Y) are significantly related. If RoS on X is significant at the low and high ends of the distribution of the independent variable within the normative range (i.e., ±2SD), there is evidence that the data support the differential susceptibility model. If RoS on X is significant at the low but not the high end of X, there is evidence that the data support the diathesis-stress model. (2) The specific measurement indexes of this method are Proportion of Interaction (PoI) and Proportion Affect (PA). PoI means the proportion that meets the difference susceptibility in the area of interaction. PoI values close to 0.50 suggest strong evidence for differential susceptibility. Values closer to 0.00 suggest strong evidence for diathesis-stress. PA is the ratio corresponding to the X value greater than the intersection of the two regression lines. Typically, the PA index will range from around 0.50 (indicating strong evidence for differential susceptibility) to 0.00 (in the clear case of diathesis-stress), and when PA≥16%, the interaction is consistent with the differential susceptibility model, and when PA≤2%, the differential susceptibility model can be rejected. (3) To test the nonlinear relationship between variables. The nonlinear relationship between X and Y may be mistaken for a differential susceptibility model, so it would be necessary to estimate differential susceptibility while demonstrating the absence of nonlinear terms X^2^ and ZX^2^. (4) When testing the effects of multiple interactions at the same time, there may be a high risk of Type I errors. Roisman et al. roposed giving priority to the sequential Bonferroni test for multiple corrections to the *p-*value [37].

## 3. Results

### 3.1. Preliminary analyses

First, the common method deviation test is carried out by Harman’s single factor test method, and the results are KMO = 0.87, Bartlett value = 27308.45, *p* < 0.001, indicating that the data can be factored analysis. Unrotated principal component factor analysis found that there are 25 common factors with a characteristic root greater than 1, and the interpretation rate of the first factor is 13.05%, so there is no common method deviation.

Means, standard deviations, and bivariate associations are shown in Table 1. As can be seen in the table, stressful life events were positively correlated with neuroticism and IGD (*r* = 0.41 and 0.36, *ps* < 0.01). Neuroticism was positively associated with IGD (*r* = 0.27, *p* < 0.01). Age was positively associated with stressful life events, neuroticism, and IGD (*r* = 0.13–0.17, *ps* < 0.01). Family economic situation was positively associated with neuroticism (*r* = 0.09, *p* < 0.01) and IGD (*r* = 0.07, *p* < 0.05).

**Table 1:** Descriptive statistics and intercorrelations between variables.

Variable	1	2	3	4	5	6	7
1. Age	1						
2. Grade	.01	1					
3. Family economic situation	-.08*	.03	1				
4. family SES	-.20**	-.11**	-.01	1			
5. Stressful life events	.17**	-.03	.01	-.06	1		
6. Neuroticism	.13**	-.02	.09**	-.05	.41**	1	
7. IGD	.15**	-.02	.07*	.02	.36**	.27**	1
*M*	14.53	1.02	2.32	10.58	1.52	2.84	0.19
*SD*	1.86	0.13	1.47	2.89	0.79	0.75	0.15

Note: *p<0.05,

**p<0.01,

***p<0.001, SES = family socioeconomic status scale, the same below.

### 3.2. Main analysis: Hierarchical multiple regression analysis

A hierarchical multiple regression analysis was conducted to evaluate the degree to which stressful life events and neuroticism affected IGD. In this analysis, the social demographic factors such as gender (boy = 1, girl = 2), age, family economic situation, and family SES were first entered, followed by stressful life events and neuroticism, and lastly, stressful life events and neuroticism interactive items.

According to Table 2, the main effects of stressful life events (*β* = 0.25, *p* < 0.001) and neuroticism (*β* = 0.18, *p* < 0.001) in predicting adolescent IGD were significant, and the interactions of stressful life events × neuroticism (*β* = 0.07, *p* < 0.05) were statistically significant.

**Table 2:** Summary of hierarchical regression of adolescent IGD.

Control variable	Online game addiction				
	*B*	*SE B*	*β*	*R* ^*2*^	*ΔR* ^*2*^
Level one					
Gender	-.26	.03	-.26***	0.1	0.1***
Age	.11	.03	.11***		
Grade	-.01	.03	-.01		
Family economic situation	.06	.03	.06		
SES	.05	.03	.05		
Second floor					
Stressful life events	.25	.03	.25***	0.22	0.22***
Neuroticism	.18	.03	.18***		
The third floor					
Stressful life events × neuroticism	.07	.03	.07*	0.23	0.22*

### 3.3. Testing for model: Analysis of Region of Significance (RoS)

**Fig 3:**
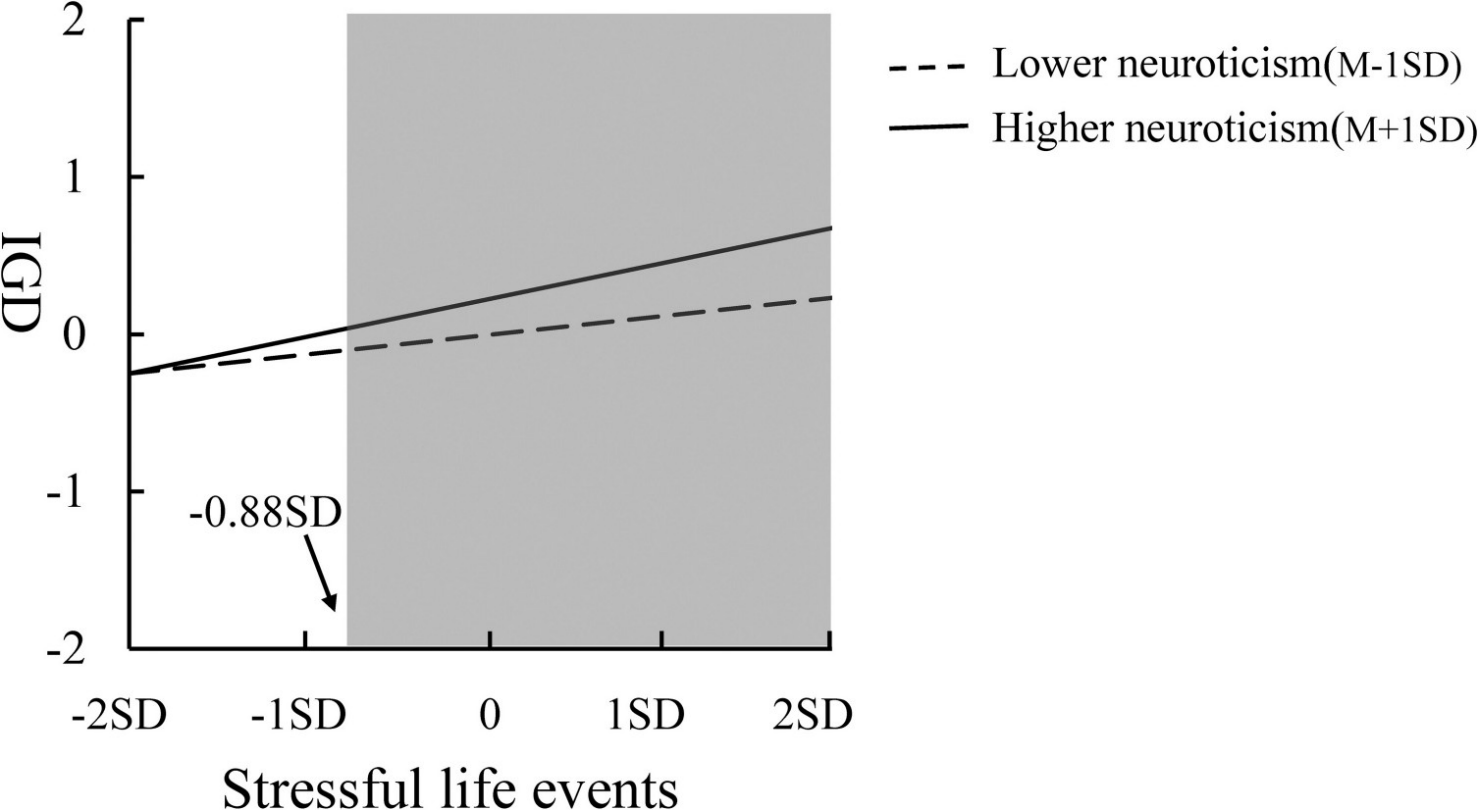
Linear regression of IGD to stressful life events. Note: gray shaded area denotes regions where the two lines significantly differ.

The regression results showed that the interaction between stressful life events and neuroticism was significant in predicting adolescent IGD, and then the RoS method was employed to further assess which theoretical model the interaction between neuroticism and stressful life events is consistent with. The results are shown in Table 3 and Fig 3. (1) The lower and upper bounds of values for the significant region of stressful life events were -15.29 and -0.88, respectively, indicating that when the value of stressful life events exceeded -0.88, the level of IGD in high neurotic adolescents was significantly higher than that in low neurotic adolescents. However, when the value of stressful life events was lower than -15.29, the level of IGD in high neurotic adolescents was significantly lower than that in low neurotic adolescents. Obviously, in this study, only the upper boundary of this significant interval was within the value range of M±2SD for stressful life events. (2) The PoI indicator refers to the proportion consistent with the differential susceptibility model in the area of interaction, which is calculated as b/(b+w). The b refers to the proportion of good results under positive environmental conditions, and W refers to the proportion of problematic behaviors under negative environmental conditions. In the present study, a low score of environmental variables represents a positive environment, while a high score represents a negative environment, which is contrary to the description of environmental variables by Roisman et al. [37]. Therefore, although the PoI’ index in this study is 1, it indicates that the proportion of good interaction effects in all interaction effects is 0. Similarly, the PA’ index represents the proportion of the area to the right of the intersection of the two regression lines. The PA’ value is 0.98, which in turn indicates that the percentage of subjects affected by good interaction effects is only 1.7%, so after clarifying the concept, the index was corrected again, and PoI = 0, PA = 0.02. Therefore, according to the indicators (1) and (2), the interaction between stressful life events and neuroticism on IGD conforms to the diathesis-stress model. The following tests in (3) and (4) are of little significance to this study, because (3) is a test for the differential susceptibility model, and the multiple interactions mentioned in (4) do not conform to the model of this study.

**Table 3:** Summary of RoS of the interaction between stressful life events and neuroticism on adolescent IGD.

Result	RoS X		PoI	PA	Intersection
	Lower boundary	Upper boundary			
Stressful life events × neuroticism	-15.29	-0.88	0	0.02	-2.13

Note: RoS X refers to the significance interval of independent variables; PoI refers to the proportion in the interaction area that is consistent with the differential susceptibility model; PA refers to the proportion of the X value greater than the intersection of the two regression lines.

## 4. Discussion

Based on general strain theory [11] and addiction theory [18], the current study examined the interaction effects of neuroticism and stressful life events on adolescent IGD. The results indicated that stressful life events was positively correlated with adolescent IGD, and the relationship between stressful life events and IGD was moderated by neuroticism. Furthermore, the interaction between stressful life events and neuroticism on IGD conformed to the diathesis-stress model. These observations expand our understanding of the complex relations between stressful life events and IGD among teenagers in China, and provide reference suggestions for the prevention and intervention of IGD in adolescents.

### 4.1. Stressful life events and adolescent internet gaming disorder

The present study examined the relationship between stressful life events and adolescent IGD. Prior studies have found that stressful life events could be positively associated with Internet addiction in adolescents [38]. The current study adds to this line of literature and reveals that stressful life events is also related to specific Internet addiction such as IGD. Our results show that stressful life events can be positively associated with adolescent IGD, which is in line with the previous research results [9]. According to the general strain theory, a variety of strains or stress experienced by adolescents might cause negative emotions, which subsequently cause problem behaviors such as IGD [11]. Specifically, stressful life events frequently cause variety of psychological strains and negative emotions on an individual while also reawakening the desire to obtain something. And the lack of reality can often be compensated in online games, resulting in online game addiction [39]. The theory of compensatory Internet use also reveals that escapism through videogames may constitute a coping strategy that is sometimes helpful [40]. Through experiments [41], researchers prove that the escapism motive is related to a preference for the virtual environment through experiments. The escapism motive could represent a dysfunctional coping strategy linked to an intense need to avoid or even dissociate from painful mental states related to current or past difficulties [42]. Thus, the more stressful life events adolescents experience, the more likely they are to lead to IGD.

### 4.2. The moderation effect of neuroticism

The present study examines neuroticism as a potential moderator between stressful life events and IGD among adolescents. The results suggest that neuroticism buffers the associations between stress life events and IGD. Specifically, adolescents with high neuroticism are more likely to develop IGD under the negative impact of higher stress life events. However, there was no significant difference in IGD levels between high and low neuroticism adolescents during low stressful life events. These results support the diathesis-stress model and are consistent with previous research [17].

It is easy to induce the problem behavior of adolescents under the double risk factors of environment and individual diathesis. The diathesis-stress model usually divides individuals into vulnerable individuals and resilient individuals, and the results of behavioral development depend on the diathesis differences in susceptibility. High neurotic individuals (vulnerable individuals) are more vulnerable to the negative impact of negative environmental and psychological behavioral problems, but in a positive environment, low neurotic individuals show the differences in psychological behavioral problems are ignored [43]. Diathesis susceptibility factors are usually hidden in consciousness, and individuals are not affected unless they are subjected to extreme stress. But once activated, they will make people’s information processing deviate from the normal processing process. Meanwhile, the increase in harmful stimulus conditions can lead victims to adopt evasive coping strategies (e.g., Internet gaming) and maintain negative emotional experiences [44]. Therefore, there is no difference in IGD among individuals with different neuroticism levels when the stressful life events are low. But individuals with high neuroticism may show more IGD when stressful life events are high. And the conclusion provides a basis for explaining the important role in personality traits between the environment and adolescent IGD. In view of the above, educators should relieve teenagers from the pressure in their family, campus, and society so as to weaken the relationship between stressful life events and adolescent IGD [9]. Especially for individuals with high neuroticism, we need to pay more attention to avoid problem behaviors such as Internet gaming disorder [20].

### 4.3. Limitations and future directions

The limitations of this study and future directions should be noted. First, this study used a cross-sectional research design. Therefore, the causal relationships cannot be inferred. Future longitudinal or experimental studies can further determine the direction of the effects. Second, the data was collected only through self-reported measures. Self-reports may be subject to increased biases (e.g., socially desirable responses) and inflated associations between antecedents and outcome variables [45]. Reports from multiple informants (e.g., parents, teachers, and peers) should be considered in future research. Third, due to the limitations of sample selection restrictions, the IGD score reported by the individual in this study is not high. By increasing the representativeness of the samples in the future, we can strengthen the external validity of the study. Fourth, in statistical methods, some studies have begun to use the emerging reparametric regression method to test the specific mode of G×E interaction from the perspective of mathematical cognition [46]. This is also a frontier topic to verify the mechanism of interaction between an individual and their environment. Finally, with the rise of behavioral genetics, many studies have found that polymorphisms of the serotonin transporter gene are associated with neuroticism, and children carrying short-position genes are more sensitive to the environment, which is significantly associated with anxiety, depression, neuroticism, and other neurotic symptoms [47]. Therefore, the susceptibility of highly neurotic adolescents to the environment may have a certain genetic basis. In the future, we can explore the deeper causes of susceptibility in adolescents from the perspective of "gene-personality-environment".

### 4.4. Implications

In this study, analysis of region of significance was used to fully and comprehensively explore the relationship between stressful life events, neuroticism, and adolescent IGD. Our findings suggest that stressful life events are associated with IGD. Thus, teachers and parents may prevent adolescent IGD and intervene in this behavior by reducing the pressure on adolescents. Moreover, our findings suggest that the positive link between high stress life events and IGD is stronger for adolescents with high neuroticism than for those with low neuroticism. Therefore, it is important to pay attention and relieve pressure on adolescents, especially those with high neuroticism.

## 5. Conclusion

Taken together, the current study reveals that stressful life events can be positively associated with adolescent IGD, and this direct effect is moderated by neuroticism. These findings support the diathesis-stress model, that is, adolescents with high neuroticism are more likely to develop IGD under the negative impact of higher stress life events. However, there was no significant difference in IGD levels between high and low neuroticism adolescents during low stressful life events.

## Supporting information

S1 Data (RAR)
